# What motivates informal carers to be actively involved in research, and what obstacles to involvement do they perceive?

**DOI:** 10.1186/s40900-021-00321-x

**Published:** 2021-11-08

**Authors:** Camilla Malm, Stefan Andersson, Maya Kylén, Susanne Iwarsson, Elizabeth Hanson, Steven M. Schmidt

**Affiliations:** 1 Department of Health and Caring Sciences, Linnaeus University, 391 82 Kalmar, Sweden; 2 Swedish Family Care Competence Centre (SFCCC), Box 681, 391 82 Kalmar, Sweden; 3 Department of Health Sciences, Lund University, P.O. Box 157, 221 00 Lund, Sweden

**Keywords:** Informal carers, Research involvement, Heterogeneity, Motivation, Obstacles

## Abstract

**Background:**

Due to demographic changes and a strained public sector operating in many countries globally, informal care is increasing. Currently, at least 1.3 million adults in Sweden regularly provide help, support and/or care to a family member/significant other. With no sign of an imminent decrease in their caring activities, it is important that informal carers are considered as a key stakeholder group within research that affects them, e.g., the co-design of carer and/or dyadic support interventions. The objective of this descriptive, quantitative study was to investigate informal carers’ perceived motivations and obstacles to become involved in research.

**Methods:**

A cross-sectional survey design was adopted, using first-wave data from a panel study. The data, collected in Sweden between September 2019 and March 2020, included survey responses from 147 informal carers who were either aged 60+ years themselves or were caring for someone who was aged 60+ years.

**Results:**

Our main results showed that informal carers are, in general, interested in research. Slightly fewer were interested in becoming actively involved themselves, but older age was the only characteristic significantly associated with less interest of being actively involved. Two latent motivational dimensions emerged from the factor analysis: ‘family motivation’ and ‘the greater good motivation’. These, according to our results, almost equally valued dimensions, described the differing reasons for informal carers to become involved in research. The most common perceived obstacle was lack of time and it was reported by more women than men.

**Conclusion:**

Our study contributes with new knowledge of informal carers’ perceived motivations and obstacles regarding carer involvement in research. Paying attention to the differing motivational dimensions held by informal carers could help researchers create conditions for more inclusive and systematic participation of informal carers within research. Thereby, increasing the opportunities for research that is deemed to be of higher societal impact. IRRID (International Registered Report Identifier): RR2-10.2196/17759.

**Supplementary Information:**

The online version contains supplementary material available at 10.1186/s40900-021-00321-x.

## Plain English summary

Around 1.3 million adults in Sweden give help, support and care to an ill, aged or disabled family member, neighbour or friend. As informal carers contribute a lot to our societies, it is important to recognise them and, where possible, to involve them in research that affects them, together with patients and professionals. The rationale for our study is the current lack of studies on carers’ views of being involved in research. 147 carers aged 60+, or who were caring for a person 60+ answered a survey that asked about carers’ motivations for and obstacles to being actively involved in research. By actively involved, we mean that participation was more than filling in a questionnaire or one single interview. We found that carers are, in general, interested in research. Slightly fewer were interested in becoming actively involved themselves. Older age was the only characteristic associated with less interest of being actively involved. A statistical test showed two motivational categories; ‘family motivation’ and ‘the greater good motivation’. These almost equally valued categories described the different reasons for carers to become involved in research. The most common obstacle was lack of time and more women than men reported this. This study adds new knowledge about carer involvement in research. The results of our study could help researchers to create a more supportive climate for actively involving carers in research. For example, by finding more flexible and creative ways to recruit carers and carry out research with them.

**Supplementary Information:**

The online version contains supplementary material available at 10.1186/s40900-021-00321-x.

## Introduction

User involvement in general has been widely recommended for several decades as a means to improve health and social service provision [1–4]. Furthermore, user involvement in research has increased in recent years [5] due to a belief that if users are involved, their insights could lead to new discoveries and more relevant and useful interventions. There are several ways users can be involved in research: as individuals or as representatives for a category of users such as patients or informal carers, as consultants providing experiential knowledge, as active collaborators working together with researchers and professionals, or as co-researchers or sponsors [6]. However, it is common to disregard distinguishing characteristics among different user categories when describing user involvement in research. Instead, they are simply aggregated under the umbrella concept ‘user’, despite previous studies having shown that the involvement of different user categories comes with specific challenges [7, 8].

Within ageing and health research, ‘informal carer’ is one of the user categories deserving of attention [9]. Informal carers often play a double role, as providers of informal care while simultaneously they may have their own need for support. Traditionally, Sweden has had a generous welfare-state system. However, due to an increasingly strained public sector, people with a need for care, help or support in their home increasingly rely on informal carers; they are commonly spouses, partners, parents, adult children or siblings, but can also constitute friends and neighbours. They provide unpaid help, support and/or care to a family member or significant other on a regular basis and usually outside of a formal or professional framework [10–13]. Although informal care is considered to be voluntary and complements public-sector services [14], many informal carers often feel that they lack a choice in the matter [15]. A caring role often affects an individual’s entire life, for example, with regards to their health and wellbeing, work life, and social inclusion [16, 17]. Hence, individualised, flexible societal support for informal carers is deemed to be highly relevant and important [18]. As a result, it is argued that the voices of informal carers must be heard and respected in all arenas that affect them, including research [16, 17, 19, 20].

The INVOLVE definition of patient and public involvement (PPI) was followed in our study, that is, *research as being carried out with or by members of the public rather than to, about or for them* (p. 2)[21]. Active involvement in research entails users as partners in the research process, instead of as research participants/subjects in a traditional sense, for example, by engaging them in advisory groups or in data collection or analysis [22]. By actively involving informal carers throughout the research process, it is recognised that new and improved strategies, which better match the identified needs and preferences of informal carers, can be developed and evaluated, leading to improved services [8]. It could be argued that findings from studies regarding other lay-user categories (e.g. members of the public, patients, service users) might be generalised to include informal carers. However, it is acknowledged that there is a need for the perspective of informal carers to be represented distinctly [5], due to the complexity embedded in the informal carer identity, which is created within a family context and in relation to another person [23].

Important goals of active user-involvement in research are inclusivity, representation, equality, non-discrimination and empowerment [24]; however, a further concern is the belief that the collective carer identity includes a certain degree of homogeneity. In reality, informal carers are a truly heterogeneous group [23], and the only thing they may have in common is—being carers [25]. Hence, it is important to address heterogeneity not only among but also within user categories to ensure valuable engagement and avoid tokenistic involvement in research [26]. Viewing carers as a homogeneous category of users may lead to non-applicable or inadequate results. Informal carers had lower response rates in medical research compared with response rates of non-carers [27], with a lack of both time and energy proposed as possible reasons [28]. In addition, some individuals do not self-identify as informal carers, even though they spend time taking care of a parent or other family member; these so-called ‘invisible carers’ are rarely involved in research and/or development work into carer issues [8, 23]. Conversely, Oldenkamp et al. [11] found higher response rates among carers who invest a large amount of time and have a heavier caregiver burden, which points to the complexity surrounding carers’ motivations for engaging in research.

Generally, reasons behind the motivation to be involved in research by different categories of the public are understudied [29]. Motivation is usually divided into two distinct types, extrinsic (external rewards) and intrinsic motivation (fx altruism, personal interest). These motivations have been shown to vary in relation to sociodemographic differences, such as gender, age, and education, and previous research findings have shown that intrinsic motivation becomes more important in older age [30]. Those who are interested in research often chose to be actively involved more than once [22, 31]. Older people face challenges and obstacles for research involvement, such as frailty, ill health and a decline in physical activities [32], which may influence and decrease their interest in and motivations more than their chronological age “per se” [22, 30].

Summing up, there is a need to look more closely into the informal carer collective and the nature and degree of interest (or otherwise) of carers in being actively involved in research. Despite the increasing awareness of the important role played by informal carers within service systems and their own needs for support, their involvement in research has rarely been explored [8, 10, 33]. Little is known about informal carers’ motivations for becoming actively involved in research or the perceived obstacles that prevent their involvement. The purpose of this study, therefore, was to investigate the views of informal carers regarding their active involvement in research, with a specific focus on motivations and obstacles. The research questions were:

- Which individual characteristics (age, gender, education, health, previous experience of active involvement in research) are associated with an interest in being actively involved in research?
- How are motivations for active involvement in research related to individual characteristics?
- What are the different latent dimensions of motivations for active involvement in research?
- What types of obstacles do informal carers report as preventing them from being actively involved in research?

## Materials and methods

This is a cross sectional study using the first data collection of an ongoing, longitudinal panel study within the UserAge research program, which engages research environments at four universities in Sweden [9]. The purpose of the panel study is to investigate the awareness and understanding of and attitudes towards user involvement in ageing and health research among different categories of users (senior citizens, informal carers, professionals) and researchers. More detailed information about the panel study is reported in the study protocol [34]. For the present study, we utilised data collected from the sub-sample of informal carers. A reporting checklist following the Guidance for Reporting Involvement of Patients and Public (GRIPP2-SF) can be found in Additional file 1 [21].

### Respondents and recruitment

Informal carers who were either caring for a person aged 60 years or more or were aged 60 years or more themselves were eligible for inclusion. The age range for inclusion was agreed on in discussions among members of a user forum [34]. As it is recognised that informal carers are often a challenging population to reach and involve in research [11, 35], we prepared alternative recruitment approaches to be used if deemed necessary. Hence, the recruitment strategy involved several steps, including referral sampling techniques [36]. Initially, a sample of 400 members of Carers Sweden (a non-governmental organisation that supports carers, independent of any political or religious affinity) were sent an invitation letter in the post, including information about the study. The response rate from this first step was just 11% (the original aim being 25%), so the research team decided to use additional recruitment channels. Information about the study was posted on the website and Facebook page of the Swedish Family Care Competence Centre, which is a centre of excellence in the field of informal care, commissioned by the National Board of Health and Welfare Sweden. In addition, the lead author (CM) contacted informal carers, professionals from municipalities (carer advisers, a carer-advocate county coordinator) and representatives from interest organisations. They were informed about the study and asked to distribute information about it to their networks and/or complete the survey themselves if applicable. In all, a total of 150 people consented to participate, with 147 people completing the survey.

### Materials

A user-forum method [37] was used to develop the questionnaire, including data collection methods and procedures. In total, eight users (senior citizens and carer organisation representatives) and three researchers participated in the three, three-hour sessions [34]. The questionnaire was piloted to assess its readability and estimate the time needed to complete the survey.

The final version of the questionnaire comprised 31 questions, including sociodemographic descriptive questions (e.g. age, gender, level of education) (Table 1). The question regarding the respondents’ level of education was dichotomised into ‘High school or less’ and ‘More than high school’. Respondents’ perception of their general health was assessed by the first question of the Short Form 36 Health Survey Questionnaire (SF-36): ‘In general, would you say your health is: excellent, very good, good, fair or poor?’ [38]. This is a reliable and valid measure of health status, as well as an important and robust predictor of multiple future health outcomes. The responses were dichotomised into ‘good’ (excellent, very good or good) and ‘poor’ (fair or poor) health.

**Table 1:** Respondent characteristics and logistic regression results (odds ratio) for interest in being actively involved in research, *N* = 147

Characteristic	Total %^1^ (*n*)	Interest in active involvement in research (*n* = 145)		OR (95% CI)
		Yes/maybe, % (*n*)	No, % (*n*)	
Age, mean (SD)	70.6 (11.2)	70.4 (11.44)	72.13 (10.50)	0.94 (0.89–0.997)
*Gender*				0.26 (0.05–1.36)
Female	84% (122)	81% (98)	19% (23)	
Male	16% (23)	91% (20)	9% (2)	
*Marital status*				0.51 (0.18–1.44)
Married/cohabitating	78% (113)	85% (94)	15% (17)	
Single	22% (32)	75% (24)	25% (8)	
*Education*				1.48 (0.52–4.25)
High-school or less	25% (36)	74% (26)	26% (9)	
More than high-school	75% (108)	85% (91)	15% (16)	
*Self-rated health*				2.54 (0.96–6.69)
Poor	37% (54)	74% (39)	26% (14)	
Good	63% (91)	88% (79)	12% (11)	
*Previously actively involved in research*				2.76 (0.80–9.52)
Yes	32% (47)	91% (42)	9% (4)	
No	68% (100)	79% (78)	21% (21)	

Missing data (*n*) = total yes, 147/147; age, 145/147; gender, 145/147; marital status, 145/147; education, 144/147; previous involvement, 147/147; health, 145/147; interest in active involvement in research, 145/147

OR, odds ratio; CI, confidence interval

^1^Valid percentage

Additional questions were developed specifically for the panel study. Thirteen questions concerned research in relation to informal caring, including a question about the respondent’s interest in research, ‘How interested are you in research about carer issues, ageing and health?’ (not at all, a little, moderately, quite a lot, very much). The response choices were dichotomised into interested (quite a lot, very much) or not interested (not at all, a little, moderately). Another question regarding interest in being actively involved in research, ‘Would you like to be actively involved in research on carer issues, ageing and health?’ (yes, no, maybe), was dichotomised as yes (yes or maybe) or no. Specifically targeting motivations and obstacles, there was one question that included 16 yes/no items. Of these, 14 items, regarding varying motivations to be actively involved in research, were included in the analysis (Table 2), while the final two, ‘nothing’ and ‘other’, were excluded. Nine yes/no items were used from a question regarding obstacles to being actively involved in research (Table 3).

**Table 2:** Response frequencies for motivational items (yes) and factor loadings for the latent motivational dimensions from the principal component analysis, *N* = 147

Motivational items and constructs for active involvement in research	Total % (n)	Mean age (SD)	Gender^1^% (n)		Education^1^% (n)		Health^1^% (n)		Factor loading
			Female	Male	More than high-school	High-school or less	Good	Poor	
*Family motivation*									
That it leads to a change in the situation of my family member	45% (65)	68.29 ^b^ (12.35)	44% (53)	48% (11)	44% (48)	43% (15)	52%^a^ (47)	32% (17)	0.681
Getting to know more about the situation of my family member	40% (58)	72.28 (10.38)	33% ^a^ (40)	74% (17)	39% (42)	43% (15)	41% (37)	37% (20)	0.561
Getting in contact with others in the same situation	36% (53)	70.85 (10.69)	36% (43)	39% (9)	35% (38)	37% (13)	37% (33)	35% (19)	0.723
That it leads to a change in my situation	31% (45)	67.69^b^ (12.62)	32% (39)	22% (5)	31% (33)	34% (12)	32% (29)	30% (16)	0.714
Getting to know more about my caring situation	29% (43)	71.19 (10.96)	29% (35)	26% (6)	32% (35)	20% (7)	31% (28)	24% (13)	0.701
Getting to know what the study will lead to	21% (30)	68.83 (9.84)	22% (26)	13% (3)	19% (21)	23% (8)	22% (20)	17% (9)	0.314
*Greater good motivation*									
The research being about something I find important	56%^2^ (81)	69.12 (11.10)	57% (69)	44% (10)	57% (61)	49% (17)	61% (55)	46% (25)	0.590
Research must move forward (i.e. someone has to do it)	52% (76)	69.91 (11.95)	49% ^a^ (59)	74% (17)	55% (59)	49% (17)	56% (50)	46% (25)	0.469
To contribute to society	40% (58)	67.43 ^b^ (12.14)	40% (48)	39% (9)	45% ^a^ (48)	26% (9)	41% (37)	39% (21)	0.743
To have better services and products	21% (31)	69.13 (9.83)	21% (25)	22% (5)	21% (23)	20% (7)	20% (18)	22% (12)	0.333
Being helpful to the researcher	19% (28)	73.32 (10.18)	17% (21)	30% (7)	23% (25)	9% (3)	21% (19)	17% (9)	0.522
I have nothing to lose	14% (21)	73.29 (7.16)	15% (18)	9% (2)	13% (14)	17% (6)	16% (14)	11% (6)	0.516
To get a sense of being important	10% (14)	69.29 (10.73)	10% (12)	9% (2)	10% (11)	9% (3)	10% (9)	7% (4)	0.456
Being prioritised for services (e.g. healthcare, social care, service, residency)	6% (8)	70.50 (12.94)	5% (6)	9% (2)	5% (5)	9% (3)	4% (4)	7% (4)	− 0.300

Missing data (*n*): total yes, 146/147; age, 144/147; gender, 145/147; education, 143/147; health, 144/147

Valid percentages used; percentages are rounded-up to whole numbers

Variance explained: 33.84

^1^Percentage within the group (gender, education, health)

^2^One person who said yes to this question did not want to state their gender

^a^Significant Chi^2^/likelihood ratio (*p* < 0.5)

^b^Significant independent-samples t-test

**Table 3:** Perceived obstacles to active involvement in research (*N* = 147)

Perceived obstacles to active involvement in research about informal carer issues, ageing and health	Yes Total % (n)	Age Mean (SD)	Gender^1^% (n)		Education^1^% (n)		Health^1^% (n)	
			Women	Men	More than high-school	High-school or less	Good	Poor
Lack of time	48% (71)^2^	66.20 ^b^ (11.45)	51% (62)	35% (8)	50% (54)	42% (15)	51% (46)	45% (24)
My own illness, disability or another hindering private situation	23% (34)	70.90 (11.80)	22% (27)	30% (7)	23% (25)	22% (8)	13%^a^ (12)	50% (27)
Too demanding for me	21% (31)	76.37 ^b^ (10.47)	18% (22)	39% (9)	18%^a^ (19)	33% (12)	21% (19)	22% (12)
Differences in expectations between researchers and myself	21% (31)	70.26 (10.50)	21% (25)	26% (6)	24% (26)	14% (5)	25% (23)	15% (8)
Difficulties in understanding the ways researchers express themselves	19% (28)	68.68 (12.90)	18% (22)	22% (5)	18% (19)	25% (9)	21% (19)	15% (8)
Researchers lack competence to involve informal carers	11% (16)	70.00 (11.74)	12% (15)	0% (0)	12% (13)	8% (3)	14%^a^ (13)	4% (2)
Differences between participants	7% (10)	77.60 ^b^ (4.33)	7% (8)	9% (2)	7% (7)	6% (2)	6% (5)	9% (5)
My lack of understanding of research	6% (9)	73.55 (10.76)	6% (7)	4% (1)	7% (8)	3% (1)	9% (8)	6% (3)
I don’t think it will lead to any changes in my situation	5% (8)	72.33 (5.79)	4% (5)	9% (2)	6% (6)	6% (2)	6% (5)	7% (4)

Missing data (*n*) = total yes, 147/147; age, 145/147; gender, 146/147; education, 144/147; health, 145/147

Valid percentage used. Percentages rounded-up to whole numbers

^1^Percentage within the group (gender, education, health)

^2^One person who said yes to this question did not want to state their gender

^a^Significant Chi^2^/likelihood ratio (*p* < 0.05)

^b^Significant independent-samples t-test

### Data collection procedure

Data were collected from August 2019 to March 2020 by Kantar Sifo, an independent survey company operating at the national level in Sweden. Depending on the recruitment method, those who were interested in participating in the study were given the option to (1) request to be contacted by a professionally trained interviewer to complete a phone survey, (2) request to receive a paper survey in the post, or (3) go directly to an online survey (the sole option for those individuals recruited through referral sampling). The majority (77%, *n* = 113) chose to complete the survey online. No reminders were sent to potential respondents, and no reward or financial incentive was offered. Data from the telephone and online surveys were directly entered into a database, and postal surveys were digitally scanned.

### Data analysis

Frequencies (percentages) and means (SD) were used to describe the respondents. The sample size varied across the analysis due to missing data for some items. Logistic regression was used, with interest in active involvement in research as the dependent variable and age, gender, education and self-rated health as independent variables. In the second step of the logistic regression, the model was adjusted for previous active involvement in research.

The Chi-square test (χ^2^) and likelihood ratio (Lχ^2^) were used to identify any relationships between each of the 14 motivation items and the respondent characteristics of gender, education and health, while independent-samples t-tests were used to identify relationships between motivation and age. When assumptions of the Chi-square test were violated, the likelihood ratio was used [39]. When assumptions of the independent-samples t-test were violated, e.g. when variances for the two groups were not equal, the alternative t-value was used [40].

A stepwise progression was used to analyse the 14 motivation items and identify latent dimensions of motivation. First, a principal component analysis (PCA) with varimax rotation was used to explore possible latent dimensions of motivation. Assumptions were met, based on the Kaiser–Meyer–Olkin measure of sampling adequacy, Bartlett’s test of sphericity and an anti-image correlation. Items with a factor loading of 0.3 or higher were considered for inclusion in each factor [40]. Factor loadings from the rotated solution, in combination with the examination of eigenvalues and scree plots, were used to determine the final number of factors/components. Additionally, four of the co-authors independently defined the components and determined item placement for any items that loaded on more than one component. Following the identification of initial latent components, confirmatory factor analysis (CFA) was used to verify the factor structure. Items loading < 0.3 were removed and the CFA was iteratively computed until all items loaded at 0.3 or higher. To assess the goodness of fit, the following criteria were used: incremental fit index (IFI) > 0.90, Tucker–Lewis Index (TLI) > 0.90, comparative fit index (CFI) > 0.90, and root mean square error of approximation (RMSEA) < 0.08 [41].

The Chi-square test was used to test for relationships between obstacles to active involvement in research in relation to the respondents’ characteristics (gender, education and health), and independent-samples t-tests were used for age. The alpha was set at *p* < 0.05 for each test. Data were analysed using IBM SPSS Statistics and AMOS (Versions 25 and 26, IBM, New York, NY, USA).

## Results

### Description of respondents

The mean age of the 147 respondents was 76.4 (SD = 11.22) years, ranging from 43 to 91 years; 84% (*n* = 122) were women. Among the sample, 113 were cohabitating (77%) and more than half (61%) were caring for a partner. A majority of respondents (83%, *n* = 121) reported an interest in research, and more than half (61%, *n* = 89) had previously participated in research studies (i.e. had been examined, tested, observed or had answered a questionnaire or participated in an interview). Fewer than half of respondents (44%, *n* = 64) were aware of the possibility to be actively involved in research, yet 47 respondents (32%) had previously been actively involved in research, assuming the role of a user. Most of the respondents (97%, *n* = 141) reported that they considered informal carers being involved in research to be important, due to their valuable experiences of informal caring. Respondent characteristics used in the further analyses are presented in Table 1.

### Interest in active involvement in research

Most respondents (83%, *n* = 120) reported that they would or might be interested in being actively involved in research about carer issues, within the field of ageing and health. Of those who had previous experience of active involvement in research, 91% (*n* = 42) were interested in being involved again.

The logistic regression showed that age was the only characteristic that was significantly associated [odds ratio (OR) = 0.94, 95% confidence interval (CI) = 0.89–0.997] with interest in active involvement in research, after also adjusting for previous active involvement in research. That is, being older was associated with a reduced interest in being actively involved in research (see Table 1 for further details).

### Motivations for involvement in research

#### Motivational items

The reasons that could motivate respondents to be actively involved in research varied. The most reported motivational item was that the research was something the respondent found important (56%, *n* = 81), closely followed by the notion that ‘someone has to do it’ to enable research to move forwards (52%, *n* = 76). Sixty-five respondents (45%) reported being motivated if research involvement would lead to a change in the situation of the cared-for person, followed by a wish to know more about the situation of the cared-for person (40%, *n* = 58).

Those who were motivated to be actively involved in research if it led to changes in the situation of their family member had a significantly lower mean age (M = 68.3, SD = 12.35) than those who did not find this reason motivating (M = 72.6, SD 9.94); t (122) = 2.27, *p* < 0.05. Those who reported being motivated to be actively involved in research if it led to changes in the caring situation were also significantly younger (M = 67.7, SD = 12.6), compared with those who were not motivated by this item (M = 72, SD = 10.38); t (72.16) = 2.01, *p* < 0.05. The item ‘to contribute to society’ was significantly more motivating for younger respondents (M = 67.43, SD = 12.14) compared with the older respondents (M = 72.83, SD = 10.13); t (107.27) = 2.79, *p* < 0.05. Men (74%, *n* = 17) were more likely than women (33%, *n* = 40) to endorse the item ‘getting to know more about the situation of my family member’ as a motivation for research involvement, Lχ^2^ (2, *n* = 145) = 14.36, *p* < 0.001. Men (74%, *n* = 17) were also more likely than women (49%, *n* = 59) to feel that ‘someone has to do it’, Lχ^2^ (2, *n* = 145) = 6.61, *p* < 0.05. Respondents who had received an education of more than high-school level (44%, *n* = 48) were more likely to report a desire to ‘contribute to society’ compared with those who had received a high-school-level education or less (26%, *n* = 9), χ^2^ (1, *n* = 143) = 3.87, *p* < 0.05. Other comparisons between motivations for active involvement and respondent characteristics (gender, education, previous experiences of involvement in research, health and age) were not significant (Table 2).

#### Latent dimensions of motivation

**Fig. 1:**
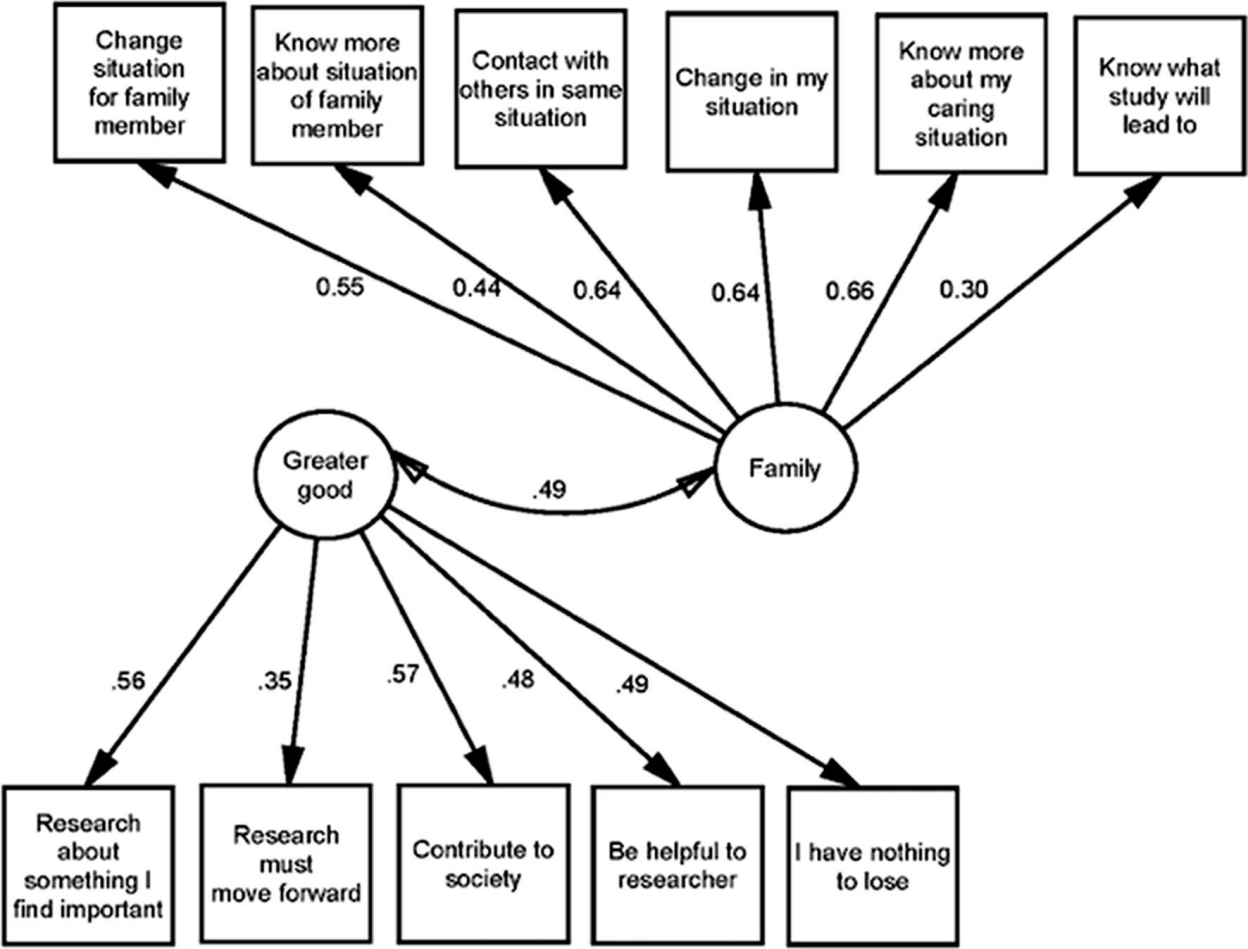
Confirmatory factor analysis (CFA) of latent motivational dimensions

In the PCA, five components had eigenvalues > 1, but following examination of the scree plots, we attempted a three-component solution. Four of the items had loadings > 0.3 on two of the three components. During the CFA, two of the three factors were highly correlated (*r* = 0.85). Hence, we proceeded to assess a two-factor solution. As shown in Table 2, all items had loadings of 0.3 or higher, and all items only loaded on one component. The two latent dimensions of motivation were defined as ‘family motivation’ and ‘greater good motivation’. CFA supported the overall two-factor solution. However, following three iterations, three items were removed from the ‘greater good dimension’ because they had factor loadings < 0.30. These were: ‘To have better services and products’, ‘Being prioritised for services’, and ‘To get a sense of being important’. In the final iteration, the family motivation dimension included six items, and the greater good motivation dimension included five items (see Fig. 1). The goodness of fit of the final structure was generally acceptable: IFI = 0.928, CFI = 0.920, and RMSEA = 0.52, but the TLI was slightly low, at 0.877. On average, 36% (*n* = 53) of participants answered yes to greater good motivation items and 34% (*n* = 49) answered yes to the family motivation items.

### Obstacles for active involvement in research

The most reported obstacle to active involvement in research, reported by nearly half of respondents, was a lack of time (48%, *n* = 71), followed by their own illness, disability or other hindering private situation (23%, *n* = 34). Others (21%, *n* = 31) thought that being actively involved in research would be too demanding for them (further details are shown in Table 3). Several reported obstacles were found to vary by age. A significantly higher proportion of older respondents considered active involvement in research was too demanding (M = 76.37, SD = 10.47) compared with younger respondents (M = 69.15, SD = 10.97); t (143) = -3.24, *p* < 0.01. Respondents who viewed differences between participants as an obstacle were significantly older (M = 77.6, SD = 4.36) compared with respondents (M = 70.13, SD = 11.41) who did not see this as an obstacle; t (20.30) = -4.439, *p* < 0.001. On the other hand, experiencing a lack of time as an obstacle was significantly more common among younger respondents (M = 66.20, SD = 11.45) compared with older ones (M = 74.79, SD 9.31); t (133.17) = 4.93, *p* < 0.001. Respondents who reported having poor health (50%, *n* = 27) were more likely to state their own illness, disability or other hindering private situation as an obstacle for active involvement in research compared with those who reported having good health (13%, *n* = 12), χ^2^(4, N = 145) = 27.83, *p* < 0.001. Other comparisons between different obstacles for active involvement in research and respondent characteristics (gender, education, earlier experiences of involvement, health and age) were not statistically significant (Table 3).

## Discussion

In addressing the lack of research describing informal carers’ active involvement in research, this study generates new knowledge on informal carers as a specific category of users and their level of interest in being actively involved in research, with a specific focus on their motivations and perceived obstacles to active involvement. Informal carers arguably share a unique perspective as providers of informal care that may simultaneously have their own support needs; therefore, their preferences are likely to differ from those of other user categories [5, 42]. Hence, it is important to listen to the voices of informal carers regarding their involvement in research.

### Interest in being involved in research

Overall, the informal carer respondents reported an interest in research. They deemed that informal carers should be actively involved in research into carer issues because of their lived experiences and practical knowledge about informal caring, a finding consistent with earlier research [4, 8, 43, 44]. However, despite their reported interest, fewer respondents were interested in being actively involved themselves. The only statistically significant variable of interest was age, where the odds of being interested in being actively involved in research became lower with increasing age. This reduced interest could in part explain why older people’s voices are, in general, less heard in comparison with other groups [45]. However, this finding contradicts that of Fudge et al. [22], who in their systematic literature review found that older people are interested in being involved in research, especially if the research findings contribute to changes in practice, for example, in services and/or policies.

### Motivations for research involvement

Initially, because the factor analysis showed a similar categorisation of motivation items to that of Lakomý et al. [29], we considered using the same labels, i.e. extrinsic and intrinsic. As an example, the item ‘That it leads to change in the situation of my family member’ was viewed as an extrinsic motivation, as it suggests that individuals choose to be involved because it leads to an external reward [46]. In turn, intrinsic motivation was seen as related to societal altruism, e.g. the item ‘To contribute to society’, referring to involvement as internally rewarding, such as by finding something interesting or enjoyable [46], or doing something because it makes oneself feel better. However, when we scrutinised the final eleven items included in the two motivational dimensions (Fig. 1), we realised that referring to the two categories as extrinsic and intrinsic motivations may not be entirely relevant. Rather, we decided to use the concept of ‘family motivation’, as presented by Menges et al. [47], for our first category. Family motivation is a type of prosocial motivation [48], defined as a desire to make extra efforts to benefit and support one’s family. It is deeply connected with one of the most fundamental values in society, caring for the family [47], and often includes both intrinsic and extrinsic motivational elements. In our interpretation, the concept includes both relational and reciprocal aspects, with kinship as a powerful driver of emotional closeness. The second category was named ‘the greater good’, because it was not clear to us that motivations for active involvement in research were for purely intrinsic reasons. As we see it, this category is about a desire to contribute to a social cause viewed as important; however, it is not explicit whether this is for intrinsic or extrinsic reasons.

The informal carers who participated in our study placed relatively similar value on both family motivation and the greater good motivation. Hence, there was a synergy between the different categories of motivational dimensions, consistent with earlier research [2], which has shown that informal carers are motivated by both a desire to contribute to the improvement of carer situations in general as well as by personal benefits [8]. This contrasts with other research, which showed that people are more motivated to be involved in research they perceive as close to their own situation [49]. If the greater good motivation were translated into altruistic reasons for involvement in research, informal carers could be viewed as motivated to become involved in research because of a wish to imbue the lives of carers with greater meaning [2]. Family motivation is likely to motivate informal carers to become actively involved in research, due to the complexity of the caring role and identity, with benefits for both the informal carer and/or the care recipient in a family context [8, 23].

Our results showed that certain characteristics were related to different motivations for active involvement in research. Older age seemed to be connected with an overall lower motivation, a result inconsistent with earlier research, which showed that intrinsic reasons for involvement increased throughout a person’s lifetime [29]. Again, this could possibly be explained by the complexity of informal caring, where earlier research has shown that the perceived caring burden develops and in many cases increases over time [23, 50]. In this regard, the finding that research involvement leading to change in their personal and in the care recipient’s situation is more important for younger informal carers is quite surprising. However, research investigating the association between age and motivation to become involved in research is sparse [30], making it difficult to compare our findings with existing empirical evidence.

The finding that gender was not related to the different motivational factor items also differs from earlier research, which has found that women are more driven by intrinsic motives for involvement in research than men [29]. Previous studies have also highlighted that informal caring affects women more negatively than it does men [15, 51], which may explain why women were more motivated by family reasons. Interestingly, men valued the family motivation factor item, ‘getting to know more about the situation of my family member’ more than women, perhaps suggesting that male informal carers are excluded more often from involvement in the care of their family member, as earlier research has suggested [52].

According to our results, 85% of the informal carers with an education of more than high-school level and 74% of those with an education level of high-school or less were interested in being actively involved in research. According to previous research, an individual’s level of education may be of importance when someone decides whether to become involved in research [29]. Moreover, our results showed that, although family motives were similar despite different levels of education, more informal carers with an education above high-school level were enticed to be involved in research for greater good reasons. This was shown by the response rates to the items ‘to contribute to society’ and ‘being helpful to the researcher’. The latter could perhaps be explained by informal carers having experienced similar situations where they had previously been involved in research projects and were therefore familiar with the challenges this may entail.

### Obstacles for active research involvement

Despite a high level of interest and motivations driving informal carers to be involved, obstacles that could hinder their active involvement in research were identified. The most common reported obstacle was a lack of time, which was also shown to be an obstacle to involvement by marginalised populations in a previous study [49]. Informal carers, particularly those carrying out extensive caring activities, may often experience a strained everyday life. Caring for a partner/spouse may occupy the main part of their day, and informal carers may not have the opportunity to either prioritise or devote their limited spare time to involvement in research [53]. We found that women perceive lack of time to be more hindering than men do. This is likely to be connected with women experiencing a more strained carer situation, with women helping a family member with personal activities related to daily living to a much greater extent than men [15, 51].

In terms of obstacles to active involvement in research, education seemed to be of importance as well. Our results revealed that those with a lower education were more likely to perceive it as too demanding, suggesting a greater difficulty to be actively involved in research. Although not comprehensively investigated in this study, earlier research has shown that low socioeconomic status, the dimension of marginalisation, and participants’ life contexts are related to greater obstacles to involvement [49]. Hence, there is a risk that the context and structure of the process of becoming involved in research itself systematically excludes some populations from becoming involved or from becoming involved in a meaningful way [54].

Our results showed that age was an important characteristic that was related to several obstacles to becoming actively involved in research. Similar to previous research, old age appears to be associated with whether an individual decides to be involved in research [55]. However, Fudge et al. argued that it is more complex an issue than simply referring to age ‘per se’ as being the main obstacle. Their study suggested that older people experience the same obstacles as younger people, such as a lack of confidence, unfamiliarity with research, medical conditions, language barriers and lack of time [22]. In our study, older informal carers were more likely to identify several of the following obstacles: lack of time, a sense that active involvement would be too demanding, and researchers’ lack of competence in engaging informal carers. Self-reported poor health was related to viewing their own illness, disability or other hindering private situation as an obstacle. This suggests that health issues, which become more common with increasing age, combined with being an informal carer, rather than age itself, may constitute important obstacles. These findings support the views of Fudge et al. [22], namely, that it might be too simplistic to state chronological age as an obstacle to active involvement in research.

It could be argued that obstacles to active carer involvement in research are possible to overcome. To do this, it is necessary to look further into and explore more flexible, creative, time and energy efficient research methods and recruitment strategies, which could help to attract a more diverse range of informal carers. However, this demands efforts to be made by researchers (and the organisations where they work), such as curiosity, respect and giving the involvement process sufficient time and resources. With regards to resources, previous research has highlighted the importance of sufficient resources to enable carers to feel valued and respected for their contributions, for example, having sufficient research funding to pay for respite care in the home so that the carer can leave the home for a few hours to participate in research activities. As well, paying salary costs to enable working carers (carers who balance paid work with informal care) to leave their paid workplace for several hours to engage in research. Resources can also usefully include transport and/or transport costs and remuneration costs to at least partially compensate carers for their time and efforts. Further, attention to seemingly small but significant practical details is highly relevant. For example, ensuring a warm and welcoming climate by providing opportunities for a lunch together prior to the start of a research activity and/or ensuring sufficient time for coffee and tea breaks with favourite snacks to stimulate interaction and the building of trusting relationships [56, 57].

Researchers also need to be aware of the often unequal and asymmetric situation between themselves and the participating informal carers [8]. Future research should explore other kinds of heterogeneity within the carer collective, including specific carer characteristics, such as who they are caring for (e.g. a parent, spouse or child), the care recipient’s illness or disability, and the length and nature of the caring relationship.

### Methodological considerations

This study has some limitations that should be mentioned. The recruitment strategy was adjusted twice to increase the number of respondents. As a result, it was not feasible to accurately determine the response rate, resulting in a potential selection bias and impeding the generalisability of the results. Therefore, this study should be considered as an illustration of possible motivations but also obstacles that may be encountered when involving informal carers in research. The data collection channels used in our study may have been more favourable to certain groups of informal carers (for example, members of a carer organisation and carers known to formal care services), as the decision to only offer the survey in an online format for those recruited through referral sampling may have hindered participation of older carers and/or carers whose voices are seldom heard. On the other hand, a recent report showed that 96% of the Swedish population (16+ years) have access to the internet [58].

More broadly, the recruitment challenges may arguably be indicative of the overall situation for informal carers generally in Sweden which has led to them being a relatively hidden group. Given the history of a generous social democratic welfare regime operating in Sweden where the State provides for its citizens from the cradle to the grave, there tends to be a low level of awareness of the existence of informal carers among society at large in general, and also by a number of health and social care professionals and decision makers in particular. This lack of awareness and recognition also exists at an individual level as many carers tend not to self-identify as carers (see Introduction above). It can be argued that this situation is mirrored by the lack of a strong, specific legislative framework for carers with comprehensive rights and supports in place regardless of the carer’s geographical location. Thus, other European countries with more liberal welfare regimes and less formal service provision for frail older and/or disabled people tend to have more statutory rights and (in theory) more extensive services for carers, together with greater recognition of informal carers within all levels of society, see for example, the UK and Ireland [59].

Additional methodological issues should also be noted. Although the survey was developed in collaboration with users (senior citizens, informal carers, professionals) and researchers [34], due to the length and phrasing of the questions, there is a risk that the survey was perceived as intimidating, causing potential respondents to choose not to respond at all, or to ‘break off’, i.e. stop completing the survey after having completed just part of it [60]. Due to the exploratory nature of this study, we chose not to adjust the level of alpha, *p* < 0.05, even though we performed a large number of statistical comparisons, which could increase the risk of a Type I error. Hence, the results of these comparisons should not be considered definitive but rather be used to generate specific hypotheses to be tested in future studies.

Nevertheless, as research conducted within the field of user involvement thus far has been dominated by small-scale, often retrospective, qualitative designs [5], designs of the type represented by the present study can be argued to comprise a valuable contribution [3, 5, 11, 34], despite their limitations. To the best of our knowledge, there have been no previous quantitative studies regarding active informal carer involvement in research. In addition to the empirical results presented, the methodological insights gained are valuable in their own right.

## Conclusion and implications

This study represents new knowledge regarding carer involvement in research, with a specific focus on the motivations for and obstacles to involvement among a sample of informal carers in Sweden. The distinction between the two types of motivation (family and greater good) can be seen to help deepen our current understanding of carer involvement in research. To date, there has been a dearth of empirical research that has explored the family as a source of motivation. An enhanced knowledge about carers’ motivations for research involvement could lead researchers to better reflect on how the impact of their research to a wider society is communicated. This, together with an increased awareness about obstacles to research involvement among carers, could enable researchers to address the prerequisites necessary for the more inclusive and systematic involvement of informal carers within research and development work that affects, or has the potential to affect, their daily lives. For example, more flexible and creative recruitment and methodological approaches that better suit carers’ individual caring situations and which in turn could enable the recruitment of more “invisible” and “hard to reach” groups of carers within research. Finally, our study highlights the importance of considering sociodemographic characteristics, health and the multifaceted identities of informal carers in order to help ensure the involvement of more diverse groups of informal carers. We consider that useful next steps, building on our study, would be to carry out a more in depth exploration of the motivational factors in relation to sociodemographic characteristics, and to delimit them so that they are actively taken into account by both researchers and practitioners alike when involving informal carers in research and/or practice development work as well as when co-designing, implementing and evaluating support programs with and for carers.

## Supplementary Information

**Additional file 1.** GRIPP2 SF.

## Data Availability

The datasets generated and analysed during this study is not publicly available due to a data use agreement between the partners involved (Lund University, Kantar SIFO, Linnaeus University), but are available from the corresponding author on reasonable request.

## Abbreviations

CFA: Confirmatory Factor Analysis
CFI: Comparative Fit Index
CI: Confidence interval
GRIPP2 SF: Guidance for Reporting Involvement of Patients and Public Short Form
IFI: Incremental Fit Index
IRRID: International Registered Report Identifier
Lχ^2^: Likelihood-ratio
M: Mean
n: Number of individuals in the sample
OR: Odds ratio
p: Probability value
PCA: Principal Component Analysis
PPI: Patient and Public Involvement
r: Correlation coefficient
RMSEA: Root Mean Square Error of Approximation
SD: Standard deviation
SF-36: Short form 36 health survey questionnaire
SFCCC: Swedish Family Care Competence Centre
TLI: Tucker Lewis Index
χ^2^: Chi Squared test
