# Supplementary material for: What motivates informal carers to be actively involved in research, and what obstacles to involvement do they perceive?
Source: Res Involv Engagem. 2021 Nov 8;7:80. doi: 10.1186/s40900-021-00321-x (PMC8574014; doi:10.1186/s40900-021-00321-x)
Supplement: Supplementary file 1 — Additional file 1. GRIPP2 SF. [file 40900_2021_321_MOESM1_ESM.docx]

**Supplementary Material.** GRIPP2 SF

| Section and topic | Item | page No |
| --- | --- | --- |
| 1: Aim | Report the aim of PPI in the study: The aim of PPI in the study was to explore the topic of PPI among informal carers’ themselves. Namely, their interest in, motivations for and obstacles to active involvement in research . | pp. 6-7 |
| 2: Methods | Provide a clear description of methods used for PPI in the study: A questionnaire was used as the data-collection method that focused on older people’s, informal carers’ and researchers’ views and experiences of active involvement in research. This study focuses purely on a quantitative analysis of informal carers’ responses to the questionnaire as opposed to the PPI related to the development of the questionnaire itself~~.~~ | pp. 7-9 |
| 3: Study Results | Outcomes—Report the results of PPI in the study, including both positive and negative outcomes | NA |
| 4: Discussion and conclusions | Outcomes—Comment on the extent to which PPI influenced the study overall. Describe positive and negative effects | NA |
| 5: Reflections/critical perspective | Comment critically on the study, reflecting on the things that went well and those that did not, so others can learn from this experience: The recruitment-channels used, such as the use of the membership register of Carers Sweden, the web-page and social media channels of SFCCC, as well personal contacs with informal carers, professionals from municipalities (carer advisers, a carer-advocate county coordinator) and representatives from interest organisations, may have been more favorable to certain groups of informal carers. Despite these issues, this study adds some methodological insights and is, with its quantitative design, a valuable contribution to the topic of informal carer involvement in research. | pp.21-22 |
